# methBLAST and methPrimerDB: web-tools for PCR based methylation analysis

**DOI:** 10.1186/1471-2105-7-496

**Published:** 2006-11-09

**Authors:** Filip Pattyn, Jasmien Hoebeeck, Piet Robbrecht, Evi Michels, Anne De Paepe, Guy Bottu, David Coornaert, Robert Herzog, Frank Speleman, Jo Vandesompele

**Affiliations:** 1 Center for Medical Genetics, Ghent University Hospital, De Pintelaan 185, 9000 Ghent, Belgium; 2 Belgian EMBnet Node, Université libre de Bruxelles, Campus de la Plaine, building NO-CP 257, Boulevard du Triomphe, 1050 Brussels, Belgium

## Abstract

**Background:**

DNA methylation plays an important role in development and tumorigenesis by epigenetic modification and silencing of critical genes. The development of PCR-based methylation assays on bisulphite modified DNA heralded a breakthrough in speed and sensitivity for gene methylation analysis. Despite this technological advancement, these approaches require a cumbersome gene by gene primer design and experimental validation. Bisulphite DNA modification results in sequence alterations (all unmethylated cytosines are converted into uracils) and a general sequence complexity reduction as cytosines become underrepresented. Consequently, standard BLAST sequence homology searches cannot be applied to search for specific methylation primers.

**Results:**

To address this problem we developed methBLAST, a sequence similarity search program, based on the original BLAST algorithm but querying *in silico *bisulphite modified genome sequences to evaluate oligonucleotide sequence similarities. Apart from the primer specificity analysis tool, we have also developed a public database termed methPrimerDB for the storage and retrieval of validated PCR based methylation assays. The web interface allows free public access to perform methBLAST searches or database queries and to submit user based information. Database records can be searched by gene symbol, nucleotide sequence, analytical method used, Entrez Gene or methPrimerDB identifier, and submitter's name. Each record contains a link to Entrez Gene and PubMed to retrieve additional information on the gene, its genomic context and the article in which the methylation assay was described. To assure and maintain data integrity and accuracy, the database is linked to other reference databases. Currently, the database contains primer records for the most popular PCR-based methylation analysis methods to study human, mouse and rat epigenetic modifications. methPrimerDB and methBLAST are available at  and .

**Conclusion:**

We have developed two integrated and freely available web-tools for PCR based methylation analysis. methBLAST allows *in silico *assessment of primer specificity in PCR based methylation assays that can be stored in the methPrimerDB database, which provides a search portal for validated methylation assays.

## Background

Alterations in the patterns of DNA methylation are among the earliest and most common events in tumorigenesis [1,2]. In the mammalian genome, methylation takes place mostly at cytosine bases that are located 5' to a guanosine in a CpG dinucleotide. While this dinucleotide is generally underrepresented in the genome, short regions are found that are rich in CpG content. Such CpG-rich regions are part of gene promoters and are coined CpG islands [3]. Both global hypomethylation and regional promoter hypermethylation have been described in a wide spectrum of cancers [4]. Hypomethylation (or absence of methylation) of CpG islands increases potential gene activity, whereas hypermethylation of these promoter-containing CpG islands is associated with decreased gene activity or silencing [5]. The development of efficient and accurate methods to study cytosine methylation is therefore of critical importance in understanding the role of DNA methylation in the development and progression of cancer. Furthermore, methylation markers open perspectives for earlier detection of malignancies and possible better prognostic assessment of the patients [6].

Several methods have been described for evaluation of cytosine methylation including digestion of DNA with methylation-sensitive restriction enzymes followed by Southern blotting or polymerase chain reaction (PCR) [7]. Southern blotting requires large amounts of high molecular weight DNA, which limits the use of this technique. The above mentioned limitations are counteracted by performing PCR, but still both methods rely on a complete enzymatic digestion of the DNA in order to prevent false-positive results.  Instead of using methylation-sensitive restriction enzymes, other methods are based on sodium bisulphite treatment of the DNA to introduce methylation-dependent sequence differences into the genomic DNA. Sodium bisulphite converts unmethylated cytosine to uracil while leaving 5-methylcytosine unchanged. Nowadays, the most frequently used DNA methylation analysis methods employ a combination of bisulphite treatment and PCR.  The methylation-sensitive single-nucleotide primer extension (Ms-SNuPE) method incorporates amplification of bisulphite-treated DNA, followed by a quantification of the ratio of methylated versus unmethylated cytosines at CpG sites [8].  An alternative method, called combined bisulphite restriction analysis (COBRA), uses standard sodium bisulphite PCR treatment followed by restriction digestion and a quantitation step [9].  A more widespread procedure combines a bisulphite treatment and PCR-single-strand conformation polymorphism analysis (Bisulphite-PCR-SSCP or BiPS) [10]. In a first step, the converted DNA is amplified with primers that have no CpG sites in the corresponding region of the original DNA, and as such amplify both unmethylated and methylated DNA. Sequence differences between amplified products from unmethylated and methylated DNA are visualised on a SSCP gel.  The fourth and one of the most popular methods is methylation-specific PCR (MSP) [11]. It heralded a breakthrough in speed and sensitivity for gene methylation analysis. After bisulphite conversion, PCR is performed using primers that distinguish methylated from unmethylated DNA. Unlike the procedures using restriction enzymes, MSP can be used to analyse any specific CpG site by appropriate primer design and it is not prone to false-positive results. MSP is very sensitive, permitting the analysis of small and heterogeneous samples, including paraffin-embedded material.  A fifth method applies the use of a sequencing strategy to analyse the methylation status a target sequence (bisulphite sequencing or BiSeq) [12]. Bisulphite converted DNA is amplified by PCR and subsequently sequenced to assess the methylation status of individual CpG's by sequence comparison with a reference sequence. A cloning step is introduced before the sequencing if the starting material contains a mixture of cells with different methylation levels.  Although the above described PCR-based DNA methylation analysis methods are easy to use, sensitive and specific, the design and experimental validation/optimisation of the primers is often difficult, labour intensive, and excludes a certain level of standardization and uniformity. To reduce the number of difficult or even unsuccessful experimental PCR optimizations, we developed methBLAST to quickly assess the specificity of a primer pair prior to the experimental evaluation step, very much like the widely accepted (or even obligated) conventional PCR primer specificity analysis using default BLAST.  Another important problem encountered during methylation analysis is the difficulty to retrieve methylation assay information for a given gene of interest by normal literature search tools. Therefore, we developed a public repository holding essential assay information (including primer sequences) for the four major PCR-based methods for DNA methylation analysis of human, mouse and rat genomes.

## Results and discussion

### methBLAST

**Figure 1:**
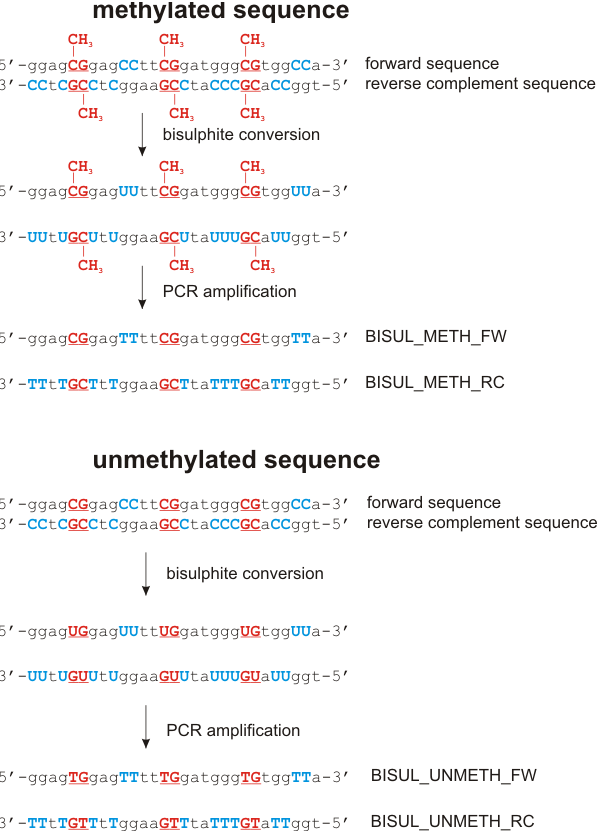
Sequence modifications during bisulphite conversion and subsequent PCR amplification of a methylated (left) or unmethylated (right) sequence. CpG dinucleotide locations are bold red and underlined; non-CpG cytosine locations are bold blue. BISUL_METH_FW, BISUL_METH_RC, BISUL_UNMETH_FW, and BISUL_UNMETH_RC are the sequence identifiers in the methBLAST databases (see Figure 2 for example).

**Figure 2:**
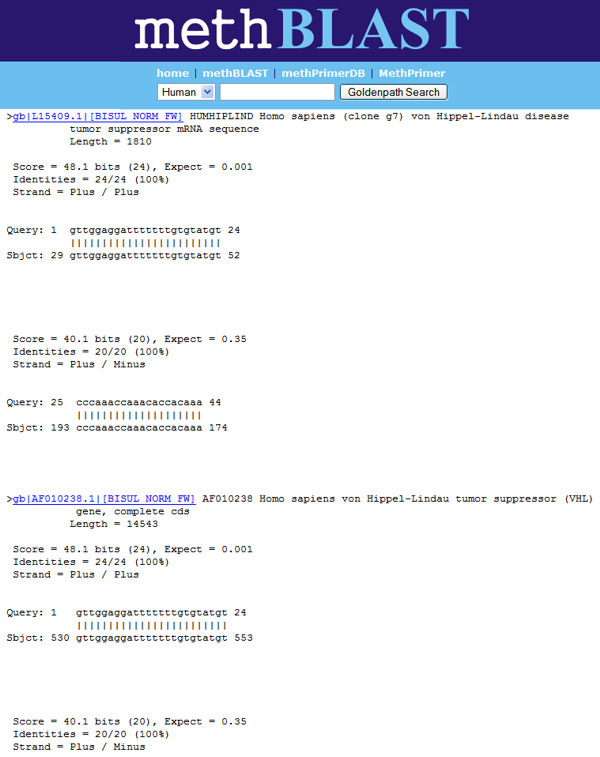
methBLAST output (top hits) showing correct alignment of an MSP primer pair for detecting unmethylated CpG's in the promoter of the human VHL gene. The tag [BISUL_UNMETH_FW] in the sequence heading indicates that it concerns a forward (FW) sequence that is bisulphite converted (BISUL) and for which the methylation status is unmethylated (UNMETH).

Performing a methBLAST search is similar to and as fast as regular BLAST [13]. The input page is divided into three parts. The first component contains a query box and two input fields for primer sequences. The query box is suited to paste a sequence in FASTA format. Primer sequence alignment can be performed by entering the forward and reverse primer sequence of an assay into the appropriate input fields. The primer sequences will be concatenated with three N's when processed by the methBLAST server. This will guarantee a correct separation of the forward and reverse sequence during the alignment step. The middle part lists the query processing options where the target species and alignment options should be selected. Only alignments against human, mouse and rat sequences from four different databases are available. The databases contain human, mouse or rat sequences from GenBank [14] for which complete CpG methylation and bisulphite modification are simulated. Because of this modification, the two daughter strands of any given sequence are no longer complementary after treatment. As either strand can serve as template for subsequent PCR amplification, we perform *in silico *bisulphite modification on both strands, assuming either an unmethylated or methylated CpG status. All cytosines (C) are replaced by thymines (T) – the DNA counterpart of uracil (U) – in sequences assumed to be completely unmethylated whereas in completely methylated sequences only the C's not followed by a G will be replaced resulting in four different sequences (methylated and unmethylated for each strand) per GenBank sequence (see Figure 1). The output format is adjustable by the options provided in the bottom section. An output window renders all relevant hits of the test sequence starting with the best alignments (see Figure 2). Depending on the database used, the sequence similarity search will be performed on either forward and reverse complement methylated (BISUL_METH_FW, BISUL_METH_RC), or forward and reverse complement unmethylated sequences (BISUL_UNMETH_FW, BISUL_UNMETH_RC). The user has to interpret the output in the same way like the BLAST output of a primer pair for normal PCR applications. A hit is only relevant if this reveals alignment of the primers at a distance close enough to generate exponential amplification. A well designed primer pair aligns exclusively with the target region, ranked high in the BLAST output. Partial alignment of the primers within a short distance on a different genomic location indicates that an assay using these primers could be aspecific and thus less reliable. Especially partial alignment of the 3' end of the primers increases the change of aspecific amplification. The methBLAST results of 14 different methBLAST searches shown in Table 1, display the differences in 'Score' and 'E value' of correct alignments which are mostly influenced by the primer length and constitution. It is impossible to use thresholds for the 'Score' and 'E value' to analyse a methBLAST output because correct alignments and misalignments show overlapping values between different primer pairs.

**Table 1:** methBLAST results of a panel of assays from methPrimerDB (1–12) and literature (13–14).

				**Best hit when both primers align on correct target**			**First misalignment**		
				
**#**	**ID**	**Assay type**	**Gene symbol**	**Sequence identifier**	**Score (bits)**	**E value**	**Sequence identifier**	**Score (bits)**	**E value**
1	14	BiPS	VHL	gb|L15409.1| [BISUL_METH_RC]	40.1 (20)	0.32	dbj|D64176.1| [BISUL_METH_RC]	32.2 (16)	79
2	236	BiPS	NKX3-1	gb|U91540.1| [BISUL_UNMETH_FW]	46.1 (23)	0.007	dbj|AP005361.2| [BISUL_UNMETH_RC]	44.1 (22)	0.026
3	26	Ms-SNuPE	RARB	emb|X56849.1| [BISUL_UNMETH_FW]	44.8 (22)	0.015	dbj|AP001217.3| [BISUL_UNMETH_RC]	40.8 (20)	0.25
4	28	Ms-SNuPE	CDKN2A	dbj|AB060808.1| [BISUL_UNMETH_RC]	46.1 (23)	0.007	emb|AL590456.5| [BISUL_UNMETH_RC]	40.1 (20)	0.41
5	13	COBRA	ESR1	gb|M69297.1| [BISUL_UNMETH_RC]	46.1 (23)	0.006	gb|AE014307.1| [BISUL_UNMETH_RC]	40.1 (20)	0.40
6	16	COBRA	FLT1	emb|AL139005.12| [BISUL_UNMETH_RC]	40.8 (20)	0.22	emb|AL731546.4| [BISUL_UNMETH_FW]	38.8 (19)	0.91
7	24	BiSeq	CDKN2C	gb|AY094608.1| [BISUL_UNMETH_FW]	50.1 (25)	4e-04	gb|AC027334.5| [BISUL_UNMETH_FW]	40.1 (20)	0.43
8	83	BiSeq	SERPINB5	gb|AC036176.8| [BISUL_UNMETH_RC]	60.0 (30)	7e-07	emb|AL133413.5| [BISUL_UNMETH_FW]	52.0 (26)	2e-04
9	1	MSP-unmeth	VHL	gb|AC018808.5| [BISUL_UNMETH_RC]	48.1 (24)	0.002	emb|AL513423.3| [BISUL_UNMETH_RC]	44.1 (22)	0.025
10	1	MSP-meth	VHL	gb|L15409.1| [BISUL_METH_FW]	44.1 (22)	0.021	gb|AC079148.9| [BISUL_METH_FW]	36.2 (18)	5.1
11	17	MSP-unmeth	CDKN2A	dbj|AB060808.1| [BISUL_UNMETH_RC]	48.1 (24)	0.002	emb|AL137127.7| [BISUL_UNMETH_RC]	42.1 (21)	0.11
12	17	MSP-meth	CDKN2A	gb|AF044170.1| [BISUL_METH_FW]	48.1 (24)	0.002	emb|AL137077.31| [BISUL_METH_RC]	36.2 (18)	6.4
13	Ref [15]	MSP-unmeth	CDKN2A	dbj|AP001626.1| [BISUL_UNMETH_RC]	38.2 (19)	1.7	emb|AL161774.49| [BISUL_UNMETH_FW]	44.1 (22)	0.028
14	Ref [15]	MSP-meth	CDKN2A	Na	Na	Na	gb|AC092171.4| [BISUL_METH_FW]	38.2 (19)	1.7

This list summarizes the output of 14 individual methBLAST searches. We randomly selected two assays from the five assay types available in methPrimerDB and listed the sequence identifier, the score and E value of the best hit when both the forward and reverse primer align correctly on the desired target and of the first alignment on an undesired target sequence. The MSP assays have two primer pairs resulting in two methBLAST outputs (9–14). All misalignment hits are the result from partial alignment of one of the primers of an assay. This does not result in aspecific amplification. The alignments of the primers from the assays submitted in methPrimerDB are correct and do not demonstrate any potentially amplifiable misalignments. Alignment results #13 and #14 were generated with primers published in literature [15] that are almost identical to the primers from methPrimerDB ID 17 for which the results are numbered as #11 and #12. Result #13 shows correct but incomplete alignment compared to result #12 and attempt #14 does not lead to any correct alignment.

Abbreviations: ID: methPrimerDB ID; BiPS: PCR-single-strand conformation polymorphism analysis; Ms-SNuPE: methylation-sensitive single-nucleotide primer extension; COBRA: combined bisulphite restriction analysis; BiSeq: Bisulphite Sequencing; MSP-unmeth: methylation-specific PCR for unmethylated target detection; MSP-meth: methylation-specific PCR for methylated target detection

Errors in primer sequences leading to incorrect alignments can be quickly identified after a methBLAST search. To demonstrate the usefulness of methBLAST we performed an MSP analysis of the CDKN2A gene using the primers and procedures published in [15]. However, we never succeeded in obtaining a PCR product (data not shown) and therefore evaluated the primers from [11] (submitted in methPrimerDB (see further) with ID 17). This assay was successful upon first attempt (data not shown) and the methBLAST outputs of both primer sets show correct alignment with the target sequence (see #11 and #12 in Table 1). On the other hand the primer sets published in [15] show only incomplete or even unsuccessful alignment (see #13 and #14 in Table 1). The forward primers of both assays are identical but the reverse primers from *Ueki et. al*. appear to contain sequence errors that caused alignment problems in methBLAST and subsequent experimental failure (see Table 2).

**Table 2:** Sequence comparison of primers from two CDKN2A MSP assays

**Ref.| methPrimerDB ID**	**Template**	**Forward primer sequence**	**Reverse primer sequence**
[11] | methPrimerDB ID 17	Unmethylated DNA	TTATTAGAGGGTGGGGTGGATTGT	CAACCCCAAACCACAACCATAA
[11] | methPrimerDB ID 17	Methylated DNA	TTATTAGAGGGTGGGGCGGATCGC	GACCCCGAACCGCGACCGTAA
[15] | -	Unmethylated DNA	TTATTAGAGGGTGGGGTGGATTGT	CAACCCCAAACC**C**ACAACCATAA
[15] | -	Methylated DNA	TTATTAGAGGGTGGGGCGGATCGC	GACCCC**C**GAACCGCGACC**C**TAA

The primer pairs from methPrimerDB ID 17 can generate a specific PCR product and show correct alignment with the target sequence when performing a methBLAST analysis (see Table 1). The primers published in [15] have identical forward primer sequences but the reverse primer sequence for detecting unmethylated DNA contains a cytosine insert between positions 12 and 13 and the reverse primer sequence for detecting methylated DNA contains a cytosine insert between positions 6 and 7 and a substitution from guanine to cytosine at position 18 (see nucleotides in bold and underlined). These sequence errors make the assay non-functional.

### methPrimerDB

If a custom designed PCR methylation assay passes the *in silico *specificity requirements (determined by methBLAST) and further experimental evaluation, submission of the assay information in methPimerDB is encouraged. In addition, authors of publications in which methylation-specific PCR, Bisulphite-PCR-SSCP, Ms-SNuPE, COBRA or BiSeq assays are developed, are kindly invited to submit their validated primer sequences. On-line data submissions are possible after free registration. During registration, personal submitter details are provided, after which an email is sent with the login name and a temporary password. By changing this password to a more convenient one, the registration is complete and the user can log in to the system and submit primer sets. For submission of large datasets, a compressed file is available in the download section of the website which contains the guidelines to complete an empty provided table with the required information.

**Figure 3:**
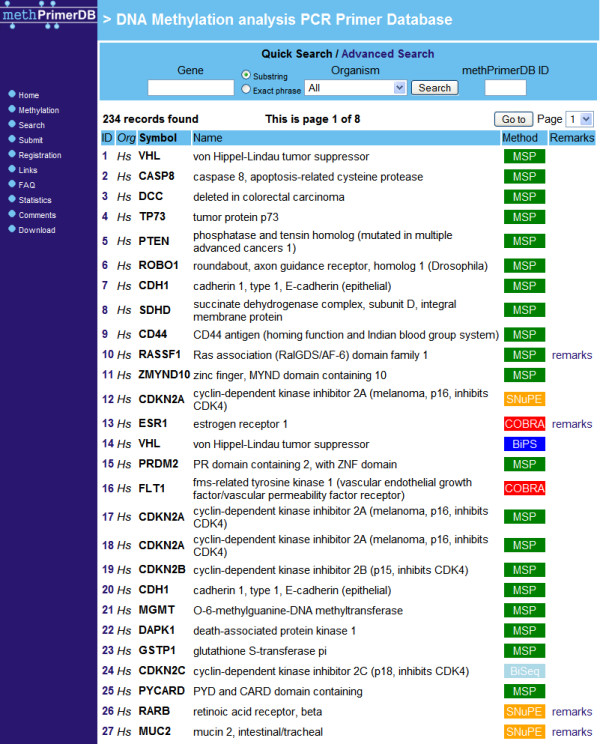
methPrimerDB search result snapshot listing the assays in a table containing the ID (direct link to the assay report), the species abbreviation, the gene symbol and name, the methylation analysis method and user remarks (if provided).

**Figure 4:**
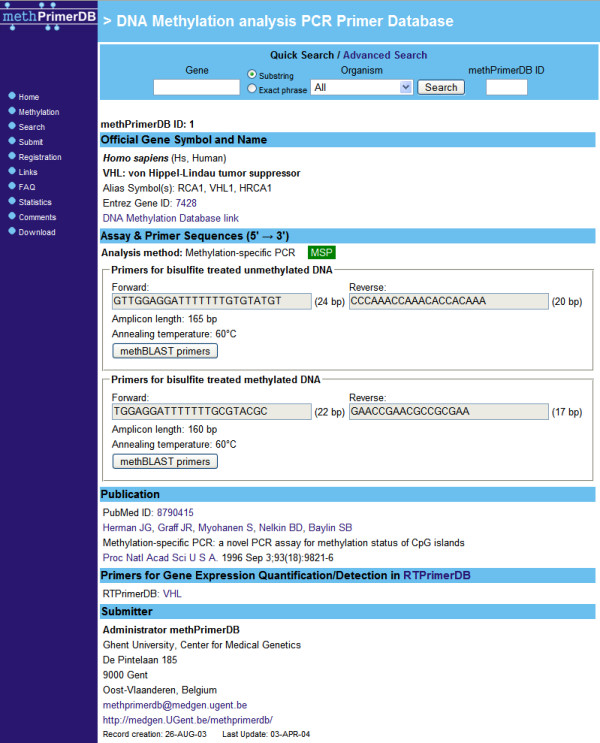
Assay report for methPrimerDB ID 1 consists of five parts containing gene annotation information, primer sequences, a publication reference, if applicable a direct link to the qPCR gene expression assay database RTPrimerDB [21] and the submitter's contact details.

New primer records should contain the official gene name, the species name, the application in which the primers are used, the nucleotide sequences of the primers, and other assay specific fields. In addition, each record provides the possibility to add submitter's remarks. Data submissions for DNA methylation analysis on human, rat and mouse are allowed, as for these organisms proper controls with respect to accuracy of the gene name fields are available via Entrez Gene [16] and the nomenclature databases for these organisms: HGNC (HUGO Gene Nomenclature Committee) [17] for human, MGD (Mouse Genome Database) [18] for mouse, and Ratmap [19] and RGD (Rat Genome Database) [20] for rat. This eliminates the presence of aliases or synonyms for official gene symbols in the database. Finally, the possibility to link the PubMed ID of an article in which the use of a PCR methylation assay is reported, makes the record more trustworthy.  The web based search engine makes it possible to query the database in different ways by type of application, organism, gene name/symbol, primer sequence, Entrez Gene ID, PubMed ID, or submitter's name.  Search results are listed as a summary of links to individual assay reports (see Figure 3). Each primer set has a unique methPrimerDB identifier to access them directly or refer to in a publication (see Figure 4).  Data integrity checks are performed during the data submission procedure. To guarantee data accuracy, the sequences in the database will be analysed on regular intervals by methBLAST search. Upon detection of possible sequence or other errors, the responsible submitter will be contacted by email.

We are planning to implement an additional feature in methPrimerDB to store the valuable feedback on assay performance from users who tested an assay from the database. The extension of the submitter's feedback section with the experimental evaluation details provided by the submitter as well as user's feedback will allow a better assessment of the quality of an individual assay. Although methPrimerDB is developed to let authors submit their own validated assays, we will populate the database in the near future with manually reviewed assays from recent literature.

## Conclusion

methBLAST and methPrimerDB are web-tools to improve the design and use of PCR-based methylation assays. A sequence homology search for methylation primers with methBLAST enables specificity assessment before experimental evaluation of a new assay. To reduce the labour-intensive design of new assays, validated methylation assays can now be stored and retrieved in methPrimerDB, a public accessible database. The database is intended to be a search portal for validated methylation assays and aims to establish a certain level of standardization and uniformity in the use of PCR based methylation assays.

## Methods

Both systems run on an Apache web server in a Linux environment. methBLAST is based on NCBI's BLAST server. The databases are generated by an in house developed Perl script (available upon request) converting a subset of the NCBI's nt database that contains all non-redundant GenBank+EMBL+DDBJ+PDB nucleotide sequences (but no EST, STS, GSS, or phase 0, 1 or 2 HTGS sequences). methPrimerDB data is stored and managed by an Oracle 9i relational database management system. The web interface to query the database is based on PHP scripts using the Oracle Call Interface (OCI). The database information and passwords are protected by the Oracle database management system which controls the access rights to the different tables.

## Availability and requirements

Free access to methBLAST  and methPrimerDB  is possible in a platform independent way by web browsers supporting image and JavaScript processing. The web sites are hosted by an Apache web server in a Linux environment. The dynamic web pages are generated by PHP scripts using the Oracle Call Interface (OCI) to connect to an Oracle database in which all methPrimerDB records and related tables are stored. methBLAST is based on NCBI's BLAST server and an in house developed Perl script is used to generate the sequence databases containing the *in silico *methylated and bisulphite treated sequences. The Perl code is available for downloading (see Additional file 1).  We welcome your feedback with respect to the methPrimerDB interface or content. You may use the feedback form available from each page or send comments to methPrimerDB@medgen.UGent.be or methBLAST@medgen.UGent.be.

## Authors' contributions

FP conceived the study, carried out the development of methPrimerDB, manages and populated the database, participated in the development of methBLAST and drafted the manuscript. JH tested the usefulness of methBLAST by experimental evaluation of MSP primers and participated in methPrimerDB data submission. PR developed and manages methBLAST and participated in the deployment of the BLAST software. EM participated in the experimental evaluation of methBLAST and participated in the methPrimerDB data submission. ADP and FS coordinated the study and provided critical input for the manuscript. GB, DC and RH carried out the deployment of the BLAST software. JV conceived the study, participated in its design and coordination and was the final editor of the manuscript.

## Supplementary Material

Additional file 1 Perl bisulphite conversion script. Perl script for creation of BLAST database files containing *in silico *methylated and bisulphite treated sequences. Click here for file
